# Elevated Red Blood Cell Distribution Width as a Poor Prognostic Factor in Patients With Hematopoietic Stem Cell Transplantation

**DOI:** 10.3389/fonc.2020.565265

**Published:** 2021-01-18

**Authors:** Xiaojiong Jia, Si Cheng, Long Zhang, Yuan Zheng, Hua Zou, Shifeng Huang, Hongxu Wang, Juan Lu, Dijiao Tang

**Affiliations:** ^1^ Department of Laboratory Medicine, The First Affiliated Hospital of Chongqing Medical University, Chongqing, China; ^2^ Department of Orthopaedics, The Second Affiliated Hospital of Chongqing Medical University, Chongqing, China; ^3^ Department of Urinary Surgery, People’s Hospital of Jiulongpo District, Chongqing, China; ^4^ Department of Otolaryngology-Head and Neck Surgery, Nanfang Hospital, Southern Medical University, Guangzhou, China

**Keywords:** red blood cell distribution width, hematopoietic stem cell transplantation, prognosis, biomarker, outcome, risk factor

## Abstract

Red cell distribution width (RDW), a measure of erythrocyte size variability, has been recently reported as an effective prognostic factor in critical illness. Hematopoietic stem cell transplantation (HSCT) has become the first choice of most patients with hematological malignancies. The aim of this study was to assess the changes of RDW in patients with HSCT and analyze the relationship between RDW and HSCT. In this study, we retrospectively enrolled 114 hematopoietic stem cell transplant patients during the period from 2015 to 2019. Logistic regression and Kaplan–Meier survival analysis were used for retrospective analysis. Multivariate analysis suggested that patients with elevated RDW (>14.5%) at three months post-transplantation have a poor clinical outcome compared with those with normal RDW ≤14.5% [odds ratio (OR) 5.12; P = 0.002]. Kaplan–Meier method analysis demonstrated that patients with elevated RDW levels (>14.5%) after hematopoietic stem cell transplantation experienced shorter progression-free survival compared to those with normal RDW levels (P = 0.008). Our study demonstrated that RDW could be an easily available and potential predictive biomarker for risk stratification in patients with HSCT. Further prospective studies are determined to confirm the prognostic value of RDW in HSCT patients.

## Introduction

Hematopoietic stem cell transplantation (HSCT) refers to a well-recognized promising procedure that treats malignant hematological diseases such as leukemia and lymphoma and restores bone marrow function in cancer patients with dysfunctional hematopoiesis, such as aplastic anemia (1). Approximately 23,000 transplants were performed each year in the United States, some of them were preceded by conditioning regimens for decreasing malignant tumor burden (2). Despite some improvements in transplantation strategies and supportive cares in recent years, transplantation still carries a significant risk for treatment-related mortality, chemotherapy-induced toxic effects, early post-transplantation complications, and even graft-*versus*-host disease (GVHD), eventually contributing to the transplant failure (3). For these reasons, there is an urgent need for novel, more effective biomarkers that can provide the opportunity for HSCT patients to receive risk-adapted therapies to improve their outcomes.

Red cell distribution width (RDW), routinely assessed as a component of complete blood count (CBC), is a quantitative index of variability for measuring the size of peripheral blood erythrocytes with higher values showing greater homogenous sizes (4). RDW is mainly used to reflect impaired erythropoiesis and abnormal red blood cell survival but correlates also with inflammation, impaired renal function, and different types of anemia, especially identifying anemia with folate and iron deficiency (5–7). Recent cumulative evidence indicates that elevated RDW was reported to be an important prognostic biomarker for increased morbidity and mortality in patients with cardiovascular diseases and chronic kidney diseases, hepatocellular carcinoma, and rheumatoid arthritis (8–11). Although RDW appears to be a powerful and independent predictor of illness severity and clinical prognosis, the mechanism for the association between RDW and outcomes remains poorly understood. It should be noted that many patients with hematopoietic stem cell transplantation are faced with several challenging risks, such as immune activation, nutritional deficiencies, impaired iron, and inadequate production of erythropoietin (EPO), and these risks may impact RDW, finally influencing the post-transplant reconstruction of the hematopoietic system (5, 12). To address this issue, we retrospectively analyzed a cohort of hematopoietic stem cell transplantation recipients with available information about RDW levels and investigated the clinical significance of RDW increment after transplantation. Moreover, the clinical outcomes were analyzed to determine if there was an association between elevated RDW and long-term prognosis.

## Materials and Methods

### Study Setting and Patients Selection

**Figure 1:**
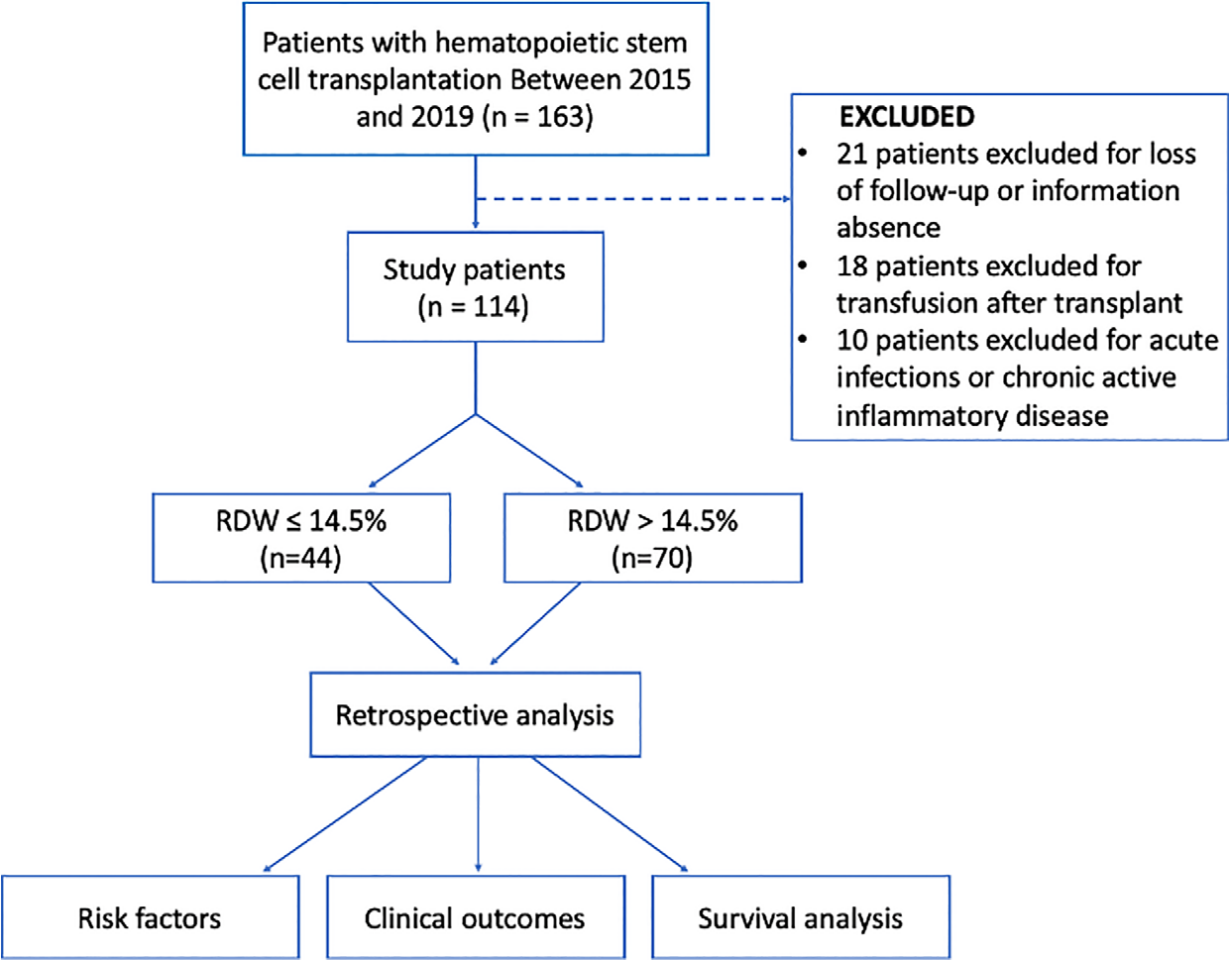
Design of this study.

After receiving approval from our institutional review board, we reviewed the electronic medical records in our retrospectively maintained database of patients with hematologic malignancies who had undergone hematopoietic stem cell transplantation from January 2015 to December 2019 in our institution and two hospital branches in Chongqing, Southwest China. The pre-operative blood cell count from the peripheral blood was available for each patient. We excluded the patients with several conditions: (i) without available data regarding RDW at transplant, (ii) those who had acute infections or chronic active inflammatory diseases, (iii) underwent blood transfusion after post-transplantation, and/or (iv) insufficient clinical and follow-up data. Finally, 114 patients were eligible for this study (**Figure 1**).

### Clinical and Laboratory Parameters

Venous blood was collected from each patient at least on admission prior to transplantation and three months after transplantation, respectively. All samples were placed in potassium ethylenediaminetetraacetic acid (EDTA-K2) anticoagulation tubes. All measurements were analyzed using XN1000 Hematology Analyzer (*Sysmex, Japan*) in which white blood cell count (WBC), hemoglobin (Hb) concentration, platelet count (PLT), mean red blood cell volume (MCV), RDW, and absolute neutrophil and lymphocyte counts were obtained directly from the blood analyzer, while albumin (Alb), alanine aminotransferase (ALT), and creatinine (CREA) were collected directly from the biochemical system database. The normal range for RDW in our hospital is defined as 11.5–14.5%.

### Potential Risk Factors

We defined a “high” RDW level when the level was >14.5%. As it shown in **Table 1**, patients were divided into two groups according to their RDW levels at three months after transplantation. Two groups were compared using several indices as potential risk factors: (i) demographics (sex and age); (ii) underlying conditions or comorbidities (hypertension, diabetes mellitus, gastrointestinal or hepatic diseases, cardiovascular diseases, and renal diseases); (iii) laboratory data [RDW, red and white blood cells (RBC and WBC, respectively) PLT, hemoglobin, mean corpuscular volume (MCV), neutrophils, lymphocytes, and pre-transplantation RDW]; (iv) transplantation related data [chemotherapy times, autologous HSC transplant, human leukocyte antigens (HLA) full matched]; (v) clinical symptoms after transplantation (sepsis, electrolyte disturbance, GVHD, hemorrhagic cystitis, hepatic and renal dysfunction, mucosal herpes, respiratory tract and urinary tract infections, digestive system diseases, hypoproteinemia); and (vi) bone marrow reconstruction (13).

**Table 1:** Baseline characteristics of study population divided by red cell distribution width (RDW) levels.

Variables	RDW ≤ 14.5%	RDW > 14.5%	P value
**Total**	**44**	**70**	
Sex (male)	30 (68.18%)	41 (58.57%)	0.328
Age (average)	32.4 ± 12.29	36.5 ± 14.38	0.168
Smoking	9 (20.45%)	23 (32.86%)	0.200
Drinking	15 (34.09%)	20 (28.57%)	0.540
**Comorbidity**			
Hypertension	1 (2.27%)	6 (8.57%)	0.246
Diabetes mellitus	2 (4.55%)	2 (2.86%)	0.642
Gastro/hepatic	10 (22.73%)	16 (22.86%)	1.000
Cardiovascular	2 (4.55%)	7 (10.00%)	0.479
Renal	2 (4.55%)	4 (5.71%)	1.000
**Laboratory Data**			
pre-transplantation RDW	13.89 ± 2.26%	15.69 ± 2.62%	**0.049**
RBC	4.01 ± 1.14	3.51 ± 1.64	**0.002**
WBC	5.03 ± 1.64	4.7 ± 1.57	0.479
PLT	149 ± 62	109 ± 55	**0.001**
HB	121 ± 18	104 ± 24	**<0.001**
MCV	93.4 ± 8.1	96.5 ± 7.8	**0.049**
Neutrophil	2.89 ± 1.64	2.42 ± 1.11	0.216
Lymphocyte	1.58 ± 0.70	1.68 ± 1.02	0.780
Alb ALT LDH CREA	43.91 ± 3.62 44.36 ± 83.45 234.52 ± 126.76 82.34 ± 64.72	39.77 ± 5.29 39 ± 49.16 361.93 ± 304 74.19 ± 24.71	**<0.001** 0.292 **0.003** 0.679
Chemotherapy (times, ≥5)	10 (22.73%)	25 (35.71%)	0.105
**Autologous HSC Transplantation**	12 (27.27%)	32 (45.71%)	**0.033**
**HLA Full Matched**	31 (70.45%)	38 (54.29%)	0.115
**Clinical symptoms**			
Sepsis	5 (11.36%)	5 (7.14%)	0.505
Electrolyte Disturbance	8 (18.18%)	19 (27.14%)	0.366
GVHD	7 (15.91%)	9 (12.86%)	0.783
Hemorrhagic Cystitis	10 (22.73%)	4 (5.71%)	**0.016**
Hepatic Dysfunction	5 (11.36%)	21 (30.00%)	**0.023**
Renal Dysfunction	3 (6.82%)	3 (4.29%)	0.675
Mucosal Herpes	18 (40.91%)	14 (20,00%)	**0.019**
Respiratory tract infection	3 (6.82%)	13 (18.57%)	0.100
Digestive system diseases	4 (9.09%)	11 (15.71%)	0.399
hypoproteinemia	2 (4.55%)	5 (7.14%)	0.149
Urinary tract infection	1 (2.27%)	7 (10.00%)	0.705
**Reconstruction**			
Myeloid	12.14 ± 3.63	11.52 ± 3.58	0.167
Megakaryocyte	16.10 ± 7.75	14.42 ± 6.28	0.322

RDW, red cell distribution width; RBC, red blood cell; WBC, white blood cell; PLT, platelets; Hb, hemoglobin; MCV, mean corpuscular volume; Alb, albumin; ALT, alanine aminotransferase; LDH, lactate dehydrogenase; CREA, creatinine; HLA, human leukocyte antigen; GVHD, graft versus host disease. Data are presented as either mean ± standard deviation, median (interquartile range), or proportions, and compared using t-, log-rank, and chi-square tests, respectively. Statistically significant p-values are shown in bold font.

### Definition

The following terms were defined prior to data analysis: pre-transplant RDW was defined as the RDW value of the patient’s blood routine at the time of admission diagnosis for transplant. Post-transplant RDW referred to the RDW value of the patient’s blood routine 3 months after HSCT. Smoking history includes patients who are smoking and those who have quit. Drinking history includes patients who are drinking alcohol and those who have stopped drinking alcohol. Sepsis was defined as a life-threatening organ dysfunction caused by host-related inflammatory response to an infection. Organic dysfunction was defined in practice as an increase in the Sequential Organ Failure Assessment (SOFA) score of at least two points from a patient’s baseline (14, 15) Graft-*versus*-host disease (GVHD) might occur after allogeneic hematopoietic stem-cell transplantation, and it was regarded as an immune response mounted against the recipient of an allograft by mature donor *αβ* T cells contained in the graft (16). Hemorrhagic cystitis was a relatively common and potentially severe complication of high-dose chemoradiotherapy, especially in conjunction with hematopoietic stem cell transplantation (17).

### Statistical Analysis

All analysis was performed using SPSS version 23.0 software. Patients were divided into two groups: (i) increased RDW levels (>14.5%) and (ii) normal RDW levels (≤14.5%). Continuous variables were present as means and standard deviations and were compared using independent sample *t*-tests. Categorical variables, of which the parameters were analyzed using *x* ^2^ tests, were presented as frequencies and percentages. Logistic regression was used to investigate the relation between the clinical outcome and laboratory or clinical data. Univariate analyses were performed separately for each of variables. Variables with P <0.10 in the univariate were included in the logistic regression model for multivariate analysis. For survival analysis, the Kaplan–Meier method was used. Log-rank test was used to estimate the statistical significance between two groups. Progression-free survival (PFS) was defined as the period from stem cell transplantation to the earliest progression of disease or death.

### Ethical Considerations

The data and the samples that were analyzed in the present study were obtained in accordance with the standards and approval of the Chongqing Medical University Institutional Review Board and Biomedical Ethics Committee. The ethics committee waived the need for written informed consent provided by participants due to the retrospective nature of the study. Because all patient data were analyzed in anonymity, no additional informed consent was required.

## Results

### Patient Characteristics

The main baseline characteristics of the 114 patients studied are listed in **Table 1**. The median age was 32 years (range = 13–68 years). Most of the enrolled patients were male (70;61.4%). According to exclusion criteria, a total of 70 patients (61.4%) with a higher RDW level (>14.5%) were included in this study. A total of 44 patients with a normal RDW level (**≤**14.5%) were used as a control group. Additionally, the mean RDW level at three months post-transplantation was 15.2 ± 2.29%.

As shown in **Table 1**, no significant difference between the two groups in the distribution of gender, age, and complications at admission was found. Compared to patients with normal RDW levels, patients with elevated RDW levels (>14.5%) generally had unfavorable laboratory results, including significantly lower levels of RBC (P = 0.002), PLT (P = 0.001), Hb (P <0.001), and Alb (P<0.001) in addition to significantly higher levels of MCV (P = 0.049) and LDH (P = 0.003). In addition, patients with high RDW levels (>14.5%) had a higher proportion of autologous stem cell transplantation (P = 0.033) and liver dysfunction after transplantation (P = 0.023), but had a lower frequency of hemorrhagic cystitis (P = 0.016) and mucosal herpes (P = 0.019) compared with the patients having normal RDW levels.

### Survival Analysis

During the median follow-up 16.5 (3–47) months period, there were 27 cases with progression or recurrence after transplantation treatment and seven deaths occurred. We defined relapse or death as a termination event. As shown in **Table 2**, univariate logistic analysis was performed on related variables to explore risk factors that may affect poor prognosis of patients with hematopoietic stem cell transplantation. RDW levels of >14.5% [odds ratio (OR) of 1.31 and 95% confidence interval (CI) 1.07–1.61; P = 0.009], WBC levels (OR 0.77; 95% CI, 0.58–1.02; P = 0.064), PLT levels (OR 0.99; 95% CI, 0.98–1.00; P = 0.004), respiratory tract infection (OR 2.77; 95% CI, 0.94–8.14; P = 0.064), and hemorrhagic cystitis after transplantation (OR 0.16; 95% CI, 0.02–1.24; P = 0.080) were proven to be potential risk factors. In the multivariate analysis (**Table 3**), elevated RDW levels (>14.5%) was demonstrated as an independent risk factors which may predict poorer prognosis for patients with hematopoietic stem cell transplantation (OR 5.12; 95% CI, 1.83–14.32; P = 0.002).

**Table 2:** Univariate analysis for progression-free survival.

Risk factor	OR (95% CI)	P- value
Male sex	0.81 (0.36–1.85)	0.62
Renal	2.48 (0.48–2.98)	0.281
pre-transplantation RDW	1.07 (0.71–1.27)	0.4
**RDW**	**1.31 (1.07**–**1.61)**	**0.009**
RBC	0.83 (0.53–1.28)	0.392
**WBC**	**0.77 (0.58**–**1.02)**	**0.064**
**PLT**	**0.99 (0.98**–**1.00)**	**0.004**
HB	0.99 (0.98–1.01)	0.479
MCV	1.01 (0.96–1.06)	0.704
Alb	0.96 (0.90–1.06)	0.525
LDH	1.00 (0.99–1.01)	0.491
Autologous HSC Transplantation	1.17 (0.51–2.65)	0.712
Sepsis	1.01 (0.25–4.16)	0.990
GVHD	1.08 (0.34–3.39)	0.893
hypoproteinemia	2 (4.55%)	5(7.14%)
**Hemorrhagic Cystitis**	**0.16 (0.02**–**1.24)**	**0.080**
Hepatic Dysfunction	1.33 (0.53–3.39)	0.544
Renal Dysfunction	0.46 (0.05–4.04)	0.48
**Respiratory tract infection**	**2.77 (0.94**–**8.14)**	**0.064**
Urinary tract infection	0.32 (0.04–2.67)	0.290

Factors related to the increased RDW (>14.9%) at post- transplantation 3 months. OR, odds ratio; CI, confidence interval; RDW, red cell distribution width; RBC, red blood cell; WBC, white blood cell; PLT, platelets; Hb, hemoglobin; MCV, mean corpuscular volume; Alb, albumin; ALT, alanine aminotransferase; LDH, lactate dehydrogenase; GVHD, graft versus host disease. Statistically significant p-values are shown in bold.

**Table 3:** Multivariate analysis for progression-free survival.

Variables	95% CI	OR	P-value
RDW	1.83–14.32	5.12	**0.002**
WBC	0.26–3.36	0.93	0.912
PLT	0.95–14.40	3.71	0.059
Hemorrhagic cystitis	0.02–1.31	0.15	0.086
Respiratory tract infection	0.67–7.79	2.29	0.185

RDW, red cell distribution width; WBC, white blood cell; PLT, platelets. Statistically significant p-values are shown in bold.

### Survival Curve

**Figure 2:**
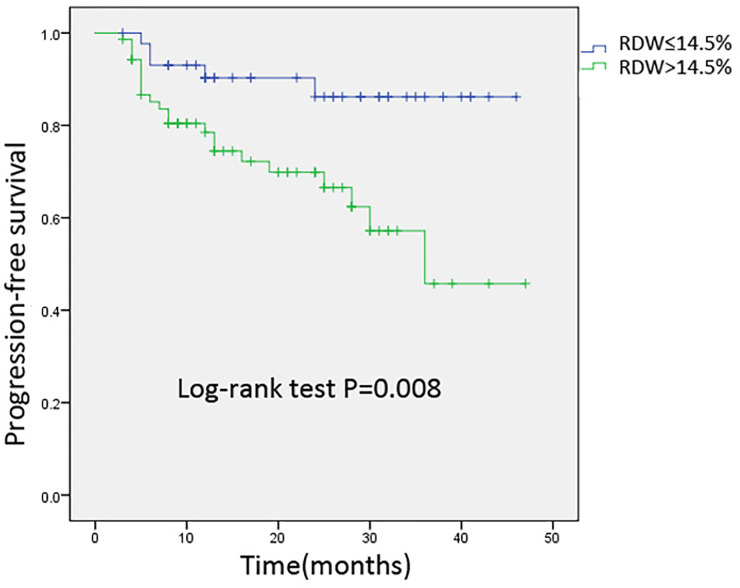
Kaplan-Meier analysis of progression-free survival for 114 patients stratified by 3-months post-transplantation RDW levels. Cumulative survival curve of the study population; y-axis indicated the cumulative survival, and x-axis indicated the months from transplantation. The green line indicates the cumulative survival of increased red cell distribution width (RDW) (>14.5%) level at three months post-transplantation; the blue line indicates the cumulative survival of others with non-elevated RDW (≤14.5%).

We used the Kaplan–Meier method to investigate the difference between the increased RDW levels (>14.5%) and normal RDW levels (≤14.5%) groups for PFS. As shown in **Figure 2**, patients with elevated RDW levels (>14.5%) after hematopoietic stem cell transplantation experienced shorter PFS compared to those without RDW levels. By using a log-rank test, it has been proven that elevated RDW levels (>14.5%) at three months post-transplantation was an independent prognostic factor for PFS (P = 0.008).

## Discussion

The present study aimed to clarify the prognostic value of baseline RDW in patients with hematopoietic stem cell transplantation. Our results demonstrated that elevated RDW levels was an independent predictor of disease progression or death after hematopoietic stem cell transplantation. Moreover, we provided the first evidence that patients with elevated RDW levels (>14.5%) after hematopoietic stem cell transplantation experienced shorter PFS compared to those with normal RDW levels. To our knowledge, this is the first report addressing the prognostic value of RDW in patients with hematopoietic stem cell transplantation.

Currently, hematopoietic stem cell transplantation is the only cure for acute leukemia, but leukemia relapse after transplantation is considered the biggest obstacle blocking the effects of transplantation, but the exact molecular mechanism of this relapse is not fully understood. Cumulative evidence indicates that several factors, such as leukemia cell tolerance to chemoradiotherapy, relapses of related gene mutations, and epigenetic abnormalities, could be associated with leukemia relapse (18). Despite new advances in transplantation strategies and supportive care, the efficacy of patients with relapse after hematopoietic stem cell transplantation is still poor. Therefore, effective monitoring and early intervention are especially important for reducing relapse rate and improving survival rate of patients with relapse after transplantation. For these reasons, an urgent need for novel, more effective biomarkers that can provide the opportunity to receive risk-adapted therapies to improve the outcome of HSCT patients exists.

RDW has been used for the differential diagnosis of anemia for decades. However, in recent years, numerous studies have found RDW to be a simple, robust, and convenient parameter associated with different human diseases. Initially, elevated RDW values were reported prognostic factors that were associated with cardiovascular mortality (19, 20). Some other studies have emphasized that elevated RDW levels can be used as an independent risk factor for poor prognosis in the hematological malignancies (21, 22). Similarly, a recent study by Yang and colleagues reported that RDW was observed to increase in colorectal cancer patients, and RDW was significantly different at each stage of colorectal cancer (23). In this present study, we focused on the prognostic value of RDW in the patients who underwent hematopoietic stem cell transplantation. Our results showed that patients with high RDW levels were more likely to have liver dysfunction after transplantation but had a lower frequency of hemorrhagic cystitis and mucosal herpes. Moreover, another important finding is the significant association between RDW and poor prognosis, specifically in hematological malignancy patients, showing RDW as a novel and powerful prognostic factor for HSCT patients.

Although the exact mechanism by which increased RDW is linked to poor prognosis for patients with HSCT is not clear, multiple factors could contribute to this association. First, elevated RDW levels may indicate impaired medullary erythropoiesis, disrupted erythrocyte metabolism, and dysregulated iron release from reticuloendothelial macrophages, thus providing opportunities for the recurrence of hematological malignancies after HSCT (24, 25). Second, inflammation could be another potential factor linking high RDW and HSCT. Some inflammatory cytokines, such as tumor necrosis alpha (TNF-α) and interleukin (IL-6), were reported to inhibit the maturation of erythrocytes through suppression of hematopoietic system in the marrow, resulting in anemia after hematopoietic stem cell transplantation (26). Third, the increased release and binding of free histones to erythrocytes increase their fragility and might contribute to the relationship between RDW and HSCT, thus finally resulting in the poor outcomes of patients with HSCT (27).

This study has some limitations. First, it was performed at our local institution with a specialized group of transplant patients, a process that potentially limits the generalizability of the results to other care settings or transplant centers. Second, the small sample size and lack of long-term follow-up prevent us from drawing a definitive conclusion about the relationship between RDW and HSCT. Third, we did not focus on some other biomarkers whether they could be dynamically correlated with RDW levels after transplantation.

In summary, this study is the first to reveal the potential predictive role of RDW in patients with HSCT. Our results will provide a new idea for reducing relapse after HSCT and improving the prognosis of patients. A more comprehensive understanding of this routine laboratory value may influence clinical decision-making and may help to improve the quality of HSCT. RDW may be used as an economical and convenient prognostic factor for the prognosis of patients with HSCT in the future.

## Data Availability Statement

The raw data supporting the conclusions of this article will be made available by the authors, without undue reservation.

## Ethics Statement

The data and the samples analyzed in the present study were obtained in accordance with the standards and approval of the Chongqing medical university institutional review board and biomedical ethics committee. The ethics committee waived the need for written informed consent provided by participants due to the retrospective nature of the study. Because all patient data were analyzed in anonymity, no additional informed consent was required.

## Author Contributions

DT and XJ designed the study, wrote the article, and consulted literature. SC, LZ, and YZ performed experiment and statistical data. HW gave key guidance for the experiment and collected the sample. JL revised manuscript. All authors contributed to the article and approved the submitted version.

## Conflict of Interests

The authors declare that the research was conducted in the absence of any commercial or financial relationships that could be construed as a potential conflict of interest.
